# The SLICE-3D dataset: 400,000 skin lesion image crops extracted from 3D TBP for skin cancer detection

**DOI:** 10.1038/s41597-024-03743-w

**Published:** 2024-08-14

**Authors:** Nicholas R. Kurtansky, Brian M. D’Alessandro, Maura C. Gillis, Brigid Betz-Stablein, Sara E. Cerminara, Rafael Garcia, Marcela Alves Girundi, Elisabeth Victoria Goessinger, Philippe Gottfrois, Pascale Guitera, Allan C. Halpern, Valerie Jakrot, Harald Kittler, Kivanc Kose, Konstantinos Liopyris, Josep Malvehy, Victoria J. Mar, Linda K. Martin, Thomas Mathew, Lara Valeska Maul, Adam Mothershaw, Alina M. Mueller, Christoph Mueller, Alexander A. Navarini, Tarlia Rajeswaran, Vin Rajeswaran, Anup Saha, Maithili Sashindranath, Laura Serra-García, H. Peter Soyer, Georgios Theocharis, Ayesha Vos, Jochen Weber, Veronica Rotemberg

**Affiliations:** 1 Dermatology Service, Department of Medicine, Memorial Sloan Kettering Cancer Center, New York, New York USA; 2 Canfield Scientific, Inc., Parsippany, New Jersey USA; 3 Frazer Institute, The University of Queensland, Dermatology Research Centre, Brisbane, Queensland Australia; 4 Department of Dermatology, University Hospital of Basel, Basel, Switzerland; 5 Computer Vision and Robotics Institute, University of Girona, Girona, Spain; 6 Melanoma Institute Australia, Sydney, Australia; 7 Faculty of Medicine and Health, University of Sydney, Sydney, Australia; 8 ViDIR Group, Department of Dermatology, Medical University of Vienna, Vienna, Austria; 9 University of Athens Medical School, Athens, Greece; 10 Dermatology Department, Hospital Clínic Barcelona, Universitat de Barcelona, IDIBAPS, Barcelona, Spain; 11 Centro de Investigación Biomédica en Red de Enfermedades Raras (CIBER ER), Instituto de Salud Carlos III, Barcelona, Spain; 12 School of Public Health and Preventive Medicine, Monash University, Melbourne, VIC Australia; 13 Victorian Melanoma Service, Alfred Hospital, 55 Commercial Road, Melbourne, VIC 3004 Australia; 14 School of Clinical Medicine, Faculty of Medicine & Health, University of New South Wales, Sydney, Australia; 15 Department of Dermatology, University Hospital of Zurich, Zurich, Switzerland; 16 FNQH Cairns Skin Cancer Clinic, Westcourt, Australia; 17 Dermatology Department, Hospital Clínic Barcelona, Barcelona, Spain

**Keywords:** Melanoma, Basal cell carcinoma, Squamous cell carcinoma, Whole body imaging

## Abstract

AI image classification algorithms have shown promising results when applied to skin cancer detection. Most public skin cancer image datasets are comprised of dermoscopic photos and are limited by selection bias, lack of standardization, and lend themselves to development of algorithms that can only be used by skilled clinicians. The SLICE-3D (“Skin Lesion Image Crops Extracted from 3D TBP”) dataset described here addresses those concerns and contains images of over 400,000 distinct skin lesions from seven dermatologic centers from around the world. De-identified images were systematically extracted from sensitive 3D Total Body Photographs and are comparable in optical resolution to smartphone images. Algorithms trained on lower quality images could improve clinical workflows and detect skin cancers earlier if deployed in primary care or non-clinical settings, where photos are captured by non-expert physicians or patients. Such a tool could prompt individuals to visit a specialized dermatologist. This dataset circumvents many inherent limitations of prior datasets and may be used to build upon previous applications of skin imaging for cancer detection.

## Background & Summary

Algorithms that can distinguish benign from malignant skin lesions with sufficient accuracy could improve triage for skin cancer detection and greatly benefit populations without access to specialized dermatologic care^1–8^. Current algorithms typically use images captured with a handheld medical device called a dermatoscope^9^, which uses a magnifying lens and a lighting source to illuminate morphologic features not otherwise visible to the naked eye^10,11^. Dermatoscopes are used commonly in dermatology clinics as an aid to evaluate skin lesions and diagnose skin cancers such as melanoma (MM)^10,12,13^, basal cell carcinoma (BCC)^14^, and squamous cell carcinoma (SCC)^15^. Dermoscopy has been integrated into the clinical practice worldwide, and a large number of images have been routinely collected for skin cancer diagnosis and monitoring. As a result, most state-of-the-art open-source skin lesion datasets for training AI models consist of dermoscopy images^16–18^. Over the past several years, researchers have explored how clinicians may benefit from utilizing dermoscopy-based AI algorithms^19–22^. However, determining which individuals should see a clinician in the first place has great potential impact^23^. Triaging applications have a significant potential to benefit underserved populations^24,25^ and improve early skin cancer detection, the key factor in long-term patient outcomes^26,27^. Similarly, decreasing unnecessary referrals can decrease delays in treating patients in true need of care and reduce burden on health systems^28,29^. With this work, we have published the SLICE-3D (“Skin Lesion Image Crops Extracted from 3D TBP”) dataset of 400,000+ standardized, de-identified, and diagnostically labeled skin images relevant to use-cases outside of specialized clinics. The image quality resembles cropped smartphone photos, which are regularly submitted by patients to their clinicians for telehealth purposes.

Contributors of this dataset are members of the International Skin Imaging Collaboration (ISIC), an international academia and industry partnership designed to reduce skin cancer morbidity and mortality through the development and use of digital skin imaging applications. ISIC engages both the dermatology and computer vision communities and works to achieve its goals by developing standards and guidelines to improve the quality, privacy, and interoperability for digital skin imaging, by making available a large and expanding open-source archive of quality labeled skin images, and by holding machine learning Grand Challenges for the computer science community in association with leading computer vision conferences. Since 2016, ISIC has hosted 5 Grand Challenges that have all been focused on developing AI for diagnostic classification using dermoscopy images^30–33^. Reader studies have demonstrated that top-performing algorithms outperform the average dermatologist on single image diagnosis in preselected lesions and limited diagnostic classes^34,35^. One prospective study tested a winning algorithm prospectively in the hands of real clinicians and in front of real patients^19^. However, these applications are confined to settings that involve the presence of a dermatologist at the time of photo capture.

Algorithms trained on more readily accessible, lower quality image types could be used by non-experts, especially in areas lacking specialized dermatologic care, for purposes of triaging concerning lesions and improving early detection^8^. Such an application might also benefit dermatologists who practice teledermatology, which has become quite common since COVID-19^23,36–39^, during which patients submit smartphone images of their skin^24^. Therefore, there is a need for algorithms to be robust with respect to image quality^40,41^. The objective for this data descriptor is to publicize a large, novel dataset and to facilitate the development of open-source AI algorithms capable of rendering diagnostic decisions from reduced quality, clinical photos resembling the resolution of smartphone images.

The images in this dataset are extracted from 3D total body photographs (3D TBP). 3D TBP is an emerging standardized imaging modality in dermatologic practice that uses a series of DSLR cameras fixed in an apparatus to capture the complete visible cutaneous surface area in one macro-quality resolution tomographic image^42–44^. Canfield Scientific, an imaging system vendor and manufacturer of a 3D TBP product^45^, has a proprietary AI-based software that identifies individual lesions on a given 3D capture^46^. This allows for the image capture and identification of all lesions on a patient, which are exportable as individual cropped photos from each lesion’s most orthogonal DSLR sensor. The SLICE-3D dataset represents every lesion from a sample of 1,000+ patients seen between 2015 and 2024 across seven high-risk dermatologic practices and three continents. Conversely, dermatologists normally use digital dermoscopy to document the more atypical lesions such as those that undergo biopsy or short-term monitoring. Utilizing 3D TBP helps circumvent the lesion-selection bias inherent to routinely collected dermoscopy image datasets^47^, where the ordinary benign examples tend to be underrepresented. Consequences from utilizing algorithms that have not been sufficiently trained on common benign lesions are uncertain.

Benign moles on an individual tend to resemble each other in terms of color, shape, size, and pattern while outlier lesions are more likely to be melanoma is an observation known as the “ugly duckling sign”^48–50^. Patient-level context is a relevant clinical consideration in addition to analysis of individual lesions for features predictive of malignancy. However, most skin lesion classification algorithms are trained for independent image analysis^51^. ISIC previously published a patient-centric dataset containing multiple dermoscopy images from a set of patients^18^, but the lesion selection bias mentioned above remained a limitation. The dataset presented here is novel because it represents each individual’s lesion phenotype more completely. This might be helpful in developing algorithms that utilize the full patient-level context.

This dataset is comprised of standardized images captured using a consistent device model and field of view (FOV). While the image resolution and detail content resemble photos captured with smartphone cameras, patient-captured images vary greatly in lighting and FOV. Standardization helps to mitigate biases that arise from differences in capture conditions specific to the image source. This is a common trait in multisource dermoscopic datasets, which typically contain source-related variations in color, lighting, and vignetting^33^. When algorithms overfit in training to features unrelated to lesion morphology, they may generalize poorly to unseen populations. Standardized photos in training may prevent overfitting and help produce robust algorithms.

## Methods

### VECTRA WB360 imaging system

VECTRA WB360 is a 3D TBP image capture system and processing software developed by Canfield Scientific that is used for documenting skin disorders. The physical device captures the entire exposed body efficiently in a single capture using an imaging pod equipped with 92 fixed cameras (46 stereo-pairs of cameras) and xenon flashes capable of both crossed-polarized and non-polarized lighting. The fully integrated DermaGraphix software allows clinicians to tag (put on record) and monitor lesions within a secure database, which can be associated with pathology reports. Clinicians can manually set tagged lesions to one of several statuses, including “biopsied/excised”. The Lesion Visualizer^52^ (LV) research tool for DermaGraphix uses AI algorithms to automatically locate lesions throughout the 3D TBP capture and estimates a set of measures for each lesion including size, shape, color, “nevus confidence”^43^, and asymmetry, among others. Hence, lesions are identified in one of two ways; (1) manual lesion tagging performed by the human, often to attribute other clinical and dermoscopy photos to the lesion; (2) automated lesion detection performed by the LV. Both forms assign a 3D coordinate to the 3D TBP image, which then guides cropping in an automated fashion.

### Lesion-image “tile” cropping

**Fig. 1:**
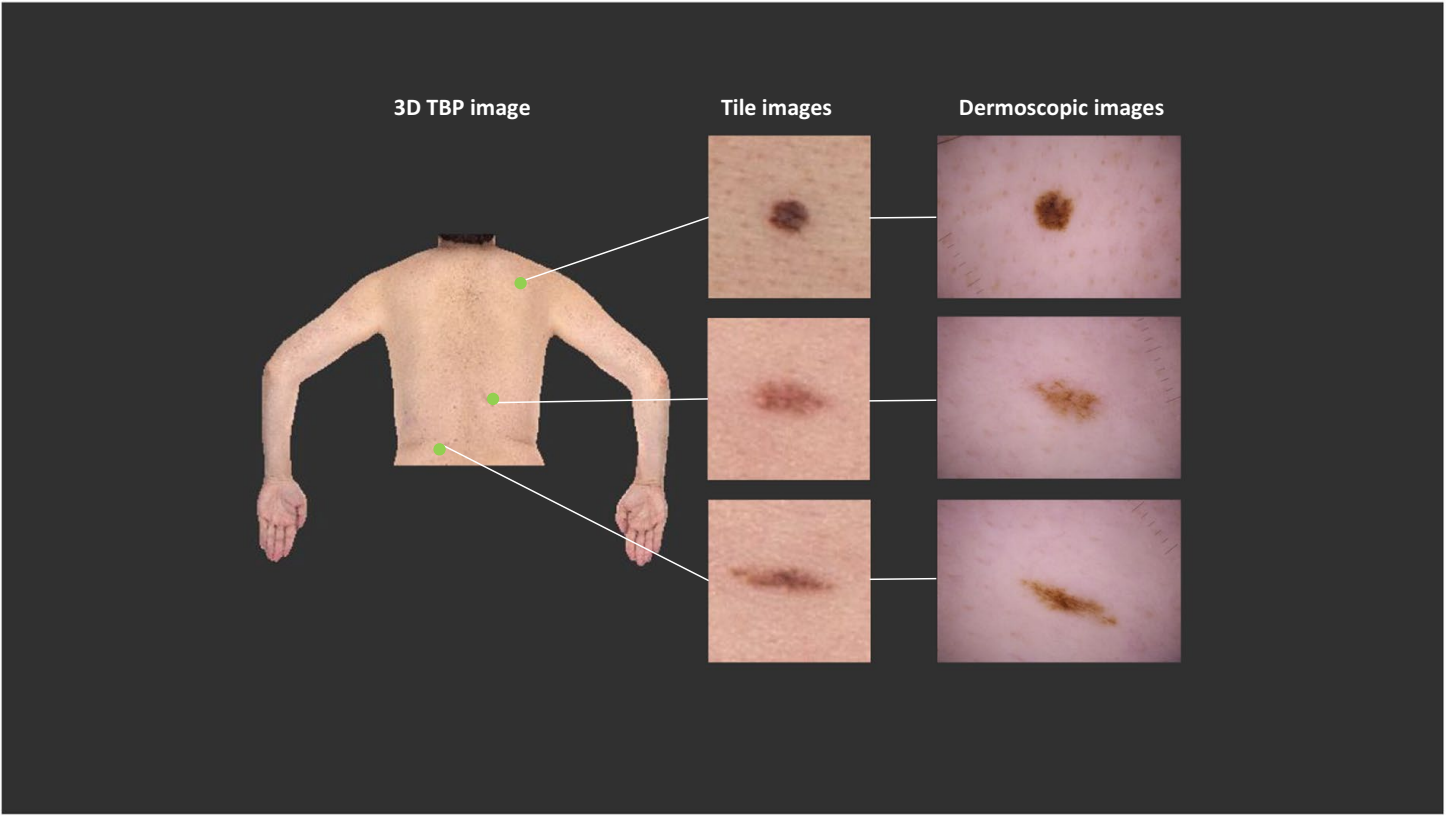
Examples of image types. The SLICE-3D dataset is comprised of tile images, which were extracted from 3D TBP images. Tile images display fewer morphologic features than dermoscopic images, which are captured in clinical settings to document and monitor specific lesions.

The ISIC2024 Tile Export Tool was developed by Canfield Scientific for the purpose of extracting standardized, de-identified, single-image photos from individual 3D TBP captures. The cropping method offers a solution to sharing TBP data for research while maintaining patient privacy and confidentiality^53–55^. The tool is capable of batch processing a list of patients. Then, for each 3D TBP capture, the tool first compares the coordinate proximities of each manual tag with each automatically detected lesion to merge pairs that represent the same lesion. Each lesion is thus represented once per 3D capture. For each lesion in the 3D TBP capture, the optimal source photo is selected as that which contains the most orthogonal view of the lesion in order to provide the least distortion in the crop. 15mm-by-15mm field-of-view cropped images centered on each lesion are exported for automatically detected lesions larger than 2.5 mm and for all manually tagged lesions regardless of size. These images are here referred to as “tiles.” Metadata files are also exported and used to associate these tiles to the 3D TBP from which each such image was derived, and include data derived from the LV software and manually tagged lesion statuses. Figure 1 shows a 2D-projection of a portion of a 3D TBP next to examples of tile images with their associated dermoscopy photos. No image preprocessing was performed on the tile images after being extracted with the ISIC2024 Tile Export Tool.

### Data collection

Plans for this dataset were initiated by the ISIC Artificial Intelligence working group and were presented at the VECTRA WB360 user group meeting during the 2023 EADV Congress. Users from seven institutions ultimately received ethics approval from their respective institutional review board (IRB) or human research ethics committee (HREC) and contributed data to ISIC for the present dataset: (1) Memorial Sloan Kettering Cancer Center, New York, USA; (2) Hospital Clínic de Barcelona, Barcelona, Spain; (3) the University of Queensland, Brisbane, Australia; (4) Medical University of Vienna, Vienna, Austria; (5) University of Athens, Athens, Greece; (6) Melanoma Institute Australia, Sydney, Australia; and (7) the University Hospital of Basel, Basel, Switzerland.

**Fig. 2:**
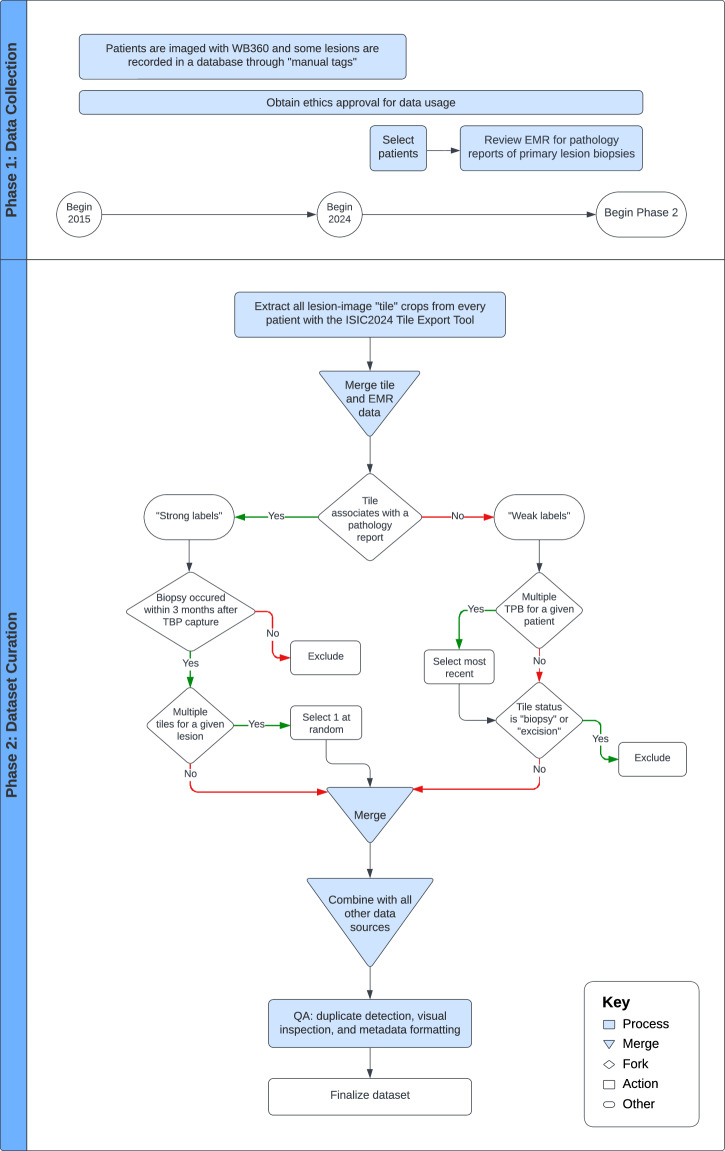
Schematic workflow of dataset curation.

Each site identified patients who had been imaged with 3D TBP between 2015 and 2024. Patients who had previously undergone a skin lesion biopsy or who were known to have been diagnosed with skin cancer were prioritized, although neither were required for inclusion. Electronic medical records (EMR) were reviewed to identify all pathology reports for skin lesion biopsies performed on the included patients. The date of each biopsy, patient- and lesion- identification numbers (from manual tags in DermaGraphix), and coded histologic diagnosis labels were recorded. The data collection process is summarized in panel 1 of Fig. 2. Tile data was exported in bulk at each site using the ISIC2024 Tile Export Tool.

### Tile sub-selection

Tiles were then sub-selected according to an algorithm, which is summarized in panel 2 of Fig. 2. The term “strong-labels” refers to tiles associated with a pathology report for a primary biopsy performed within 3 months of the 3D TBP capture, while “weak-labels” refers to tiles assumed benign based on clinical evaluation, which were not associated with a reported biopsy. Tiles associated with a pathology report prior to or after 3 months of imaging were excluded. Also excluded were tiles that were not associated with a pathology report but whose status was set to “biopsied/excised”, as their diagnostic label could not be found.

Weak-label tiles were selected from each patient’s most recent 3D TBP capture containing examples of manual tags. The newest 3D TBP capture was chosen if the patient had no manual tags. When multiple qualifying strong labeled tiles were available for a given biopsied lesion (due to multiple 3D TBPs) one was selected at random.

### Quality assurance

Protective health information (PHI) was scraped from metadata files and replaced with coded patient identifiers. All tile images underwent a manual review process in which examples of PHI were flagged and removed, in addition to occasionally included examples of non-lesions (e.g., knuckles, nipples, bellybuttons, tattoos) that were erroneously identified by the lesion detection software. Images underwent two duplicate detection measures. First, exact pixel information was compared across all possible image pairs to confirm that no exact duplicates were present. As a second measure, near duplicates to the strong-labeled images were identified and removed. All image embeddings were extracted using the MONET model published by Kim *et al*.^56^. Every strong-labeled image and its weak-labeled nearest neighbor were reviewed. If both images represented the same lesion, the weak-labeled example was excluded. Near-duplicates were removed in less than 5% of pairs and could occur in instances where the software failed to merge the manual tag with the automatically detected lesions based on 3D coordinate proximities due primarily to imprecise manual tag placement.

### Obtaining ethics approvals for data sharing

Under the ISIC terms-and-conditions^57^, a contributor agrees to upload only images to which they have been duly authorized by their own institution to transit and license to data users in accordance with the ISIC terms and one of three Creative Commons licenses selected by the contributor: CC-0, CC-BY, or CC-BY NC. Accordingly, the data from each institution has been discretely licensed for use in this dataset in accordance with what is permitted by the respective institutional IRB and/or HREC.

Memorial Sloan Kettering Cancer Center, under IRB protocol 16–974, contributed anonymized data collected under clinical image capture and consent was waived due to the anonymized nature of the images. This data subset is provided under CC-BY.

Data from the Department of Dermatology, Hospital Clínic, University de Barcelona were compiled from patients who previously had given written consent for the use of anonymized images for clinical and scientific purposes. The research ethics committee of the Hospital Clínic de Barcelona approved (HCB/2023/0213) the licensing of their data for inclusion in the dataset. This data subset is provided under CC-BY NC.

Images from the University of Queensland Dermatology Research Centre were collected from a subset of participants from two studies conducted at the Princess Alexandria Hospital, Brisbane, Australia who provided written informed consent to share de-identified images through open access research databases. The two studies, ‘Mind Your Moles’ Study^58^ and Health Outcomes Programs Study^45^, were approved by Metro South Health HREC (HREC/16/QPAH/125 and HREC/17/QPAH/816) and University of Queensland HREC (2016000554 and 2018000074), respectively. The ‘Mind Your Moles’ study^58^ was additionally approved by Queensland University of Technology (Brisbane, Australia) (1600000515), and QIMR Berghofer Medical Research Institute (Herston, Australia) (P2271). This data subset is provided under CC-BY.

The images from the Department of Dermatology, Medical University of Vienna were acquired from total body photographs of patients who were photographed with one of the total body photography systems used at the Department of Dermatology (Vectra-3D, Fotofinder 2D, conventional photography) and who have given their written consent for the use of pseudonymised images for scientific purposes. This was approved by the ethics committee of the Medical University of Vienna, Austria (1996/2023). This data subset is provided under CC-BY NC.

Data from Andreas Sygros Hospital for Cutaneous and Venereal Diseases, University of Athens were compiled from patients who previously had given written consent for the use of anonymized images for clinical and scientific purposes. The research ethics committee of Andreas Sygros Hospital approved the licensing of this data to Memorial Sloan Kettering Cancer Center for inclusion in the dataset through the data transfer agreement MSK.DTA.0000.0582. This data subset is provided under CC-BY.

Melanoma Institute Australia received approval (X20-0241 and 2020/ETH01411: Melanoma Image Annotation and Analysis Collaboration) from the Sydney Local Health District HREC to share data accrued for registered trial ACTRN12619001706167 (ACEMID) approved by the Metro South HREC under protocol HREC/2019/QMS/57206. All participants provided written consent for ACEMID, and separate consent was provided for use of images and other clinical data in research projects. This data subset is provided under CC-0.

Data from the University Hospital Basel were acquired during a clinical trial approved by the local ethics committee in Switzerland (2020-02482) and registered with ClinicalTrials.gov (NCT04605822). All patients who contributed to this dataset provided optional written informed consent for both publication and transferring of their data and images to other databases for analysis in Switzerland and abroad. This data subset is provided under CC-BY NC.

## Data Records

### Data accessibility

Note that the project contains two versions of the dataset: SLICE-3D and SLICE-3D Permissive. Both versions are stored on the ISIC Archive. SLICE-3D, comprising all data from the seven institutions, is permanently available at 10.34970/2024-slice-3d^59^. Note that SLICE-3D is released under a Creative Commons non-commercial attribution (CC-BY NC) license due to the agreed licensing terms and conditions of the providing institutions. Four of seven institutions provided data under either an attribution license (Memorial Sloan Kettering Cancer Center, the University of Queensland, and the University of Athens; CC-BY) or no rights reserved (Melanoma Institute Australia; CC-0), which are not limited to non-commercial use. This subset, called SLICE-3D Permissive, is distinctly accessible at 10.34970/2024-slice-3d-permissive^60^ in perpetuity and is released under the CC-BY license. Future changes to the ISIC Archive will not affect the versions of the dataset, as described here, accessible at the listed DOIs.

### Record description

**Fig. 3:**
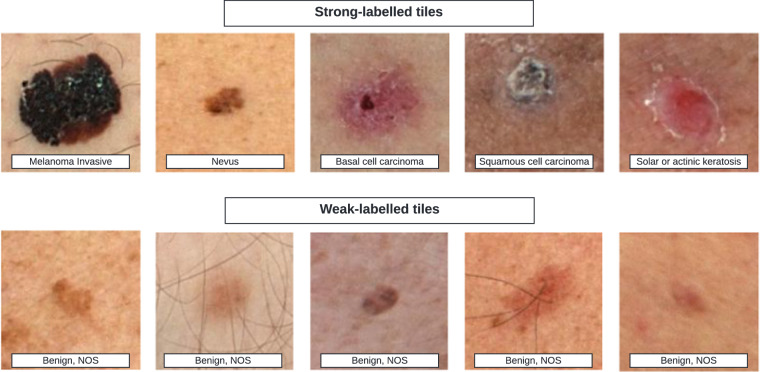
Example tiles of varying diagnostic classes.

This dataset consists of diagnostically labeled images (tiles). Each example consists of a.jpg file and associated metadata, including a diagnosis label and relevant attributes, patient sex and age at time of imaging, anatomic location of the lesion, the lighting modality of the 3D TBP capture, data from the Lesion Visualizer (e.g., estimate of lesion diameter), and a coded patient identification number for attributing separate tiles to the same patient. Tiles that were associated with a manual tag also contain a unique lesion-identification number. More information on each metadata field is listed in Table 1. Distributions for the overall dataset are presented in Table 2. Image files depict individual skin lesions with a 15mm-by-15mm field-of-view and average image size of 133px-by-133px. Examples of tiles of diagnostic classes are shown in Fig. 3.

**Table 1:** Data element dictionary.

Field(s)	Description
isic_id	Filename without the file extension. This is the unique image ID from the ISIC Archive.
iddx_full, iddx_1, …, iddx_5	Fully classified lesion diagnosis. First- through fifth-level diagnosis.
attribution	For usage and licensing purpose, but also synonymous with the medical center from which the image was sourced.
copyright_license	Creative commons license attributed to the image.
patient_id	Unique patient identifier from the ISIC Archive.
age_approx	Approximate age of patient at time of imaging
Sex	Sex of the patient
lesion_id	Unique lesion identifier from the ISIC Archive. Presence of this element indicates it was a manual tag.
anatom_site_general	ISIC Archive variable for location of a lesion on the body*.
clin_size_long_diam_mm	Maximum diameter of the lesion (mm)*.
mel_mitotic_index	Mitotic index of invasive malignant melanomas.
mel_thick_mm	Thickness in depth of melanoma invasion.
image_type	Structured field of the ISIC Archive for image type.
tbp_tile_type	Lighting modality of the 3D TBP source image.
tbp_lv_L, tbp_lv_A, tbp_lv_B, tbp_lv_C	L, A, B, Chroma inside lesion*.
tbp_lv_Lext, tbp_lv_Aext, tbp_lv_Bext, tbp_lv_Cext	L, A, B, Chroma outside lesion*.
tbp_lv_deltaL, tbp_lv_deltaA, tbp_lv_deltaB	Average L,A,B contrast (inside lesion vs. outside lesion)*.
tbp_lv_H	Hue inside the lesion; calculated as the angle of A* and B* in L*A*B* color space. Typical values range from 25 (red) to 75 (brown)*
tbp_lv_Hext	Hue outside the lesion*.
tbp_lv_areaMM2	Area of lesion (mm^2^)*.
tbp_lv_area_perim_ratio	Border jaggedness, the ratio between lesions perimeter and area. Circular lesions will have low values; irregular shaped lesions will have higher values. Values range 0–10*.
tbp_lv_color_std_mean	Color irregularity, calculated as the variance of colors within the lesion’s boundary. Lesions which are relatively flat or even in color will have low values, while lesions containing multiple different colors will have higher values. Unlike color asymmetry, this is not concerned with spatial distribution. This score is calculated in L*A*B* color space and considers the standard deviation of intensity values within each color channel. Values range 0–10*.
tbp_lv_deltaLBnorm	Contrast between the lesion and its immediate surrounding skin. Low contrast lesions tend to be faintly visible such as freckles; high contrast lesions tend to be those with darker pigment. Calculated as the average delta L*B* of the lesion relative to its immediate background in L*A*B* color space. Typical values range from 5.5 to 25*.
tbp_lv_dnn_lesion_confidence	Lesion confidence score (0–100 scale)*.
tbp_lv_Eccentricity	Eccentricity*.
tbp_lv_Location	Classification of anatomical location, divides arms & legs to upper & lower; torso into thirds*.
tbp_lv_location_simple	Classification of anatomical location, simple*.
tbp_lv_minorAxisMM	Smallest lesion diameter (mm)*.
tbp_lv_nevi_confidence	Nevus confidence score (0–100 scale) is a convolutional neural network classifier estimated probability that the lesion is a nevus. The neural network was trained on approximately 57,000 lesions that were classified and labeled by a dermatologist^*,^^43^.
tbp_lv_norm_border	Border irregularity (0–10 scale); the normalized average of border jaggedness and asymmetry*.
tbp_lv_norm_color	Color variation (0–10 scale); the normalized average of color asymmetry and color irregularity*.
tbp_lv_perimeterMM	Perimeter of lesion (mm)*.
tbp_lv_radial_color_std_max	Color asymmetry, a measure of asymmetry of the spatial distribution of color within the lesion. This score is calculated by looking at the average standard deviation in L*A*B* color space within concentric rings originating from the lesion center. Values range 0–10*.
tbp_lv_stdL	Standard deviation of L inside lesion*.
tbp_lv_stdLExt	Standard deviation of L outside lesion*.
tbp_lv_symm_2axis	Border asymmetry; a measure of asymmetry of the lesion’s contour about an axis perpendicular to the lesion’s most symmetric axis. Lesions with two axes of symmetry will therefore have low scores (more symmetric), while lesions with only one or zero axes of symmetry will have higher scores (less symmetric). This score is calculated by comparing opposite halves of the lesion contour over many degrees of rotation. The angle where the halves are most similar identifies the principal axis of symmetry, while the second axis of symmetry is perpendicular to the principal axis. Border asymmetry is reported as the asymmetry value about this second axis. Values range 0–10*.
tbp_lv_symm_2axis_angle	Lesion border asymmetry angle*.
tbp_lv_x, tbp_lv_y, tbp_lv_z	Coordinates of the lesion on 3D TBP*.

*Canfield Scientific, Inc. Lesion Visualizer metric^52^.

**Table 2:** Frequency distributions across contributing sources.

Characteristics	Overall		Athens	Barcelona	Basel	Brisbane	New York	Sydney	Vienna
***Image subset***									
**Total**	**401,059**		**7,976**	**105,724**	**65,218**	**51,768**	**129,068**	**28,665**	**12,640**
Unique lesions	401,059	100.0%	7,976	105,724	65,218	51,768	129,068	28,665	12,640
Manual tags	22,058	5.5%	167	1,720	3,004	9,056	6,212	1,792	107
***Diagnosis***									
**Malignant**	**393**	**0.1%**	**6**	**72**	**13**	**81**	**174**	**33**	**14**
Adnexal epithelial proliferations - Follicular (BCC)	163		—	23	2	45	70	14	9
Epidermal proliferations (SCC)	73		—	7	—	26	39	1	—
Melanocytic proliferations (Melanoma)	157		6	42	11	10	65	18	5
*Melanoma in situ*	80		*2*	*17*	—	*7*	*35*	*16*	*3*
*Melanoma invasive*	*63*		*4*	*24*	—	*1*	*30*	*2*	*2*
*Other/NOS*	*14*		—	*1*	*11*	*2*	—	—	—
**Indeterminate**	**114**	**0.0%**	**1**	**12**	**2**	**60**	**39**	—	—
Epidermal proliferations (AK)	39		—	3	1	21	14	—	—
Melanocytic proliferations	75		1	9	1	39	25	—	—
**Benign**	**400,552**	**99.9%**	**7,969**	**105,640**	**65,203**	**51,627**	**128,855**	**28,632**	**12,626**
Adnexal epithelial proliferations - Apocrine or Eccrine	1		—	1	—	—	—	—	—
Adnexal epithelial proliferations - Follicular	2		1	—	—	—	1	—	—
Epidermal proliferations	83		—	5	2	37	38	—	1
Melanocytic proliferations	443		13	67	128	31	168	31	5
Fibro-histiocytic	15		—	1	1	6	7	—	—
Vascular	3		—	1	—	—	2	—	—
Cysts	2		—	1	—	1	—	—	—
Flat melanotic pigmentations - not melanocytic nevus	5		—	—	—	1	3	—	1
NOS	399,991		7,955	105,564	65,072	51,551	128,629	28,601	12,619
***Anatomic site***									
Head/neck	12,046	3.0%	353	3,416	2,320	1,533	3,229	809	386
Anterior torso	87,770	21.9%	1,774	23,546	14,075	7,806	31,525	5,722	3,322
Posterior torso	121,902	30.4%	2,842	32,725	19,822	13,569	40,495	8,366	4,083
Upper extremity	70,557	17.6%	1,475	18,536	11,312	9,676	22,225	5,310	2,023
Lower extremity	103,028	25.7%	1,532	27,332	15,600	16,841	30,441	8,458	2,824
Unknown	5,756	1.4%	—	169	2,089	2,343	1,153	—	2
***TBP Lighting***									
White light	115,156	28.7%	—	1	484	126	114,545	—	—
XP light	285,903	71.3%	7,976	105,723	64,734	51,642	14,523	28,665	12,640
***Patient subset***									
**Total**	**1,042**		**16**	**163**	**230**	**176**	**398**	**44**	**15**
**Sex**									
Male	551	52.9%	9	75	115	87	229	26	10
Female	458	44.0%	7	87	88	84	169	18	5
Unknown	33	3.2%	—	1	27	5	—	—	—
***Approximate age***									
[−20)	8	0.8%	—	—	6	1	1	—	—
[20–25)	16	1.5%	1	2	5	1	7	—	—
[25–30)	28	2.7%	—	4	5	2	17	—	—
[30–35)	56	5.4%	2	4	16	10	23	1	—
[35–40)	59	5.7%	—	3	21	11	22	1	1
[40–45)	107	10.3%	2	10	24	20	43	7	1
[45–50)	85	8.2%	—	17	20	16	27	4	1
[50–55)	119	11.4%	5	26	23	17	40	5	3
[55–60)	133	12.8%	2	16	35	26	48	4	2
[60–65)	122	11.7%	2	21	21	21	46	9	2
[65–70)	129	12.4%	—	18	16	32	56	5	2
[70–75)	76	7.3%	1	12	18	12	29	3	1
[75–80)	46	4.4%	—	12	9	1	21	3	—
[80–85)	33	3.2%	1	10	5	—	14	2	1
[85+)	12	1.2%	—	4	2	1	4	—	1
Unknown	13	1.2%	—	4	4	5	—	—	—

The diagnostic labels conform to a consensus taxonomy for skin neoplasms developed in a Delphi process involving dermatologic experts^61^. While strong-labeled images contain descriptive precise lower-level terms, weak-labeled images are labeled “Benign”, a branch in the first layer of the model.

## Technical Validation

Lesions were identified on 3D TBP images in an automatic fashion and exported as individual lesion crops. The existence of a primary lesion in each image was confirmed by visual inspection.

The entire malignant diagnostic class consists of strong-labels because their ground truth was confirmed through retrospective review of pathology reports and histopathology is considered the gold standard for diagnosing skin lesions^62,63^. The malignant class included lesions diagnosed as either melanoma, basal cell carcinoma, or squamous cell carcinoma within 3 months of 3D TBP capture. Melanoma *in situ* and invasive melanoma were both coded as melanoma.

The benign diagnostic class contains both strong-labels and weak-labels because, while some were attributable to a pathology report dated within 3 months of 3D TBP capture, most never underwent a skin biopsy. Tiles that were never attributed to a pathology report and were not tagged by the clinician as biopsied/excised were assumed benign NOS. All patients in this dataset were treated per standard of care and the decision to biopsy any given lesion was based on clinician discretion. The vast majority of skin lesions are non-cancerous^64^. The standard of care when a clinician is presented with a concerning lesion is to perform a biopsy^62^. The weak-label tiles in this dataset depict lesions that were viewed by a dermatologist during a full body skin exam and were not determined to be concerning.

## Usage Notes

The field of view of each tile is constant (15 × 15 mm), but the pixel resolution is variable due to varying angles and distances between the patient’s skin and the DSLR sensors in the apparatus. Users of the dataset should use relative pixel distance (as opposed to absolute pixel distance) when conducting image analysis. In addition, the metadata element clin_size_long_diam_mm approximates the maximum diameter of the lesion. However, this measurement is most robust on lesions with clearly outlined boundaries and less conclusive in presence of sun damaged skin. Like the other metadata fields derived from the LV feature, this measurement is an approximation made with an AI model, which serves as a benchmark.

Each tile is derived from either a cross-polarized light (XP) or a non-polarized white light 3D TBP. Lighting modality is the predominant technical factor influencing variability in hue, tint, tone, and shade between the otherwise standardized images contained in this dataset. Thus, the lighting modality evident in the metadata element tbp_tile_type should be acknowledged.

There are no exact duplicate tiles in the dataset, and each corresponds to a unique lesion. When lesions are closely adjacent, however, they may be visible toward the boundary of one another’s tile. The metadata of each tile corresponds to the point-of-interest lesion present nearest to the center of the frame.

Numerous tiles can be associated to the same patient with the metadata element patient_id. Interaction of patient_id and body location fields such as anatom_site_general, location, or location_simple may be used to fine cluster the dataset for similar contextual analyses observed by clinicians^51^, such as the ugly duckling sign. Lesions in these clusters tend to exhibit similarities due to patient phenotypes and like sun exposure^65^.

While effort was made to recruit populations at high risk for skin cancer around the world, it was not possible to document the skin tone of the individuals in this dataset due to the retrospective nature of the collection. Dataset users should be aware of this limitation.

The SLICE-3D dataset was used as the training dataset for the ISIC 2024 Grand Challenge. Additional data from each of the seven contributors (1–7) was collected for a separate test set, not described here, for evaluating results of that competition. Two other sites provided data that was allocated entirely to the test set: (8) Alfred Hospital, Melbourne, Australia; and (9) FNQH Cairns, Westcourt, Australia. While the methods of curating the SLICE-3D dataset and the ISIC 2024 test set were consistent, the test set is beyond the scope of this data descriptor.

## Data Availability

Custom generated code used by all sites for collating the raw data from the ISIC2024 Tile Export Tool, which is described in the Methods section, is available at https://github.com/ISIC-Research/2024-challenge-dataset. All code is written in Python 3.10 and utilizes commonly used open-source packages, all of which are available on pypi.org.
